# Revealing Sexual Dimorphism in Prolactin Regulation From Early Postnatal Development to Adulthood in Murine Models

**DOI:** 10.1210/jendso/bvad146

**Published:** 2023-11-28

**Authors:** Alejandra Abeledo-Machado, Milagros Peña-Zanoni, Dana Bornancini, Graciela Díaz-Torga

**Affiliations:** Instituto de Biología y Medicina Experimental (IBYME), Fundación IBYME, CONICET, Buenos Aires 1428, Argentina; Instituto de Biología y Medicina Experimental (IBYME), Fundación IBYME, CONICET, Buenos Aires 1428, Argentina; Instituto de Biología y Medicina Experimental (IBYME), Fundación IBYME, CONICET, Buenos Aires 1428, Argentina; Instituto de Biología y Medicina Experimental (IBYME), Fundación IBYME, CONICET, Buenos Aires 1428, Argentina

**Keywords:** prolactin, prolactinomas, postnatal development, sex differences, TGFβ1, activins

## Abstract

Serum prolactin (PRL) levels exhibit a gradual rise both in male and female rats from birth to adulthood, with females consistently displaying higher levels compared to age-matched males. This pattern has traditionally been attributed to the development and maturation of endocrine and neuroendocrine networks responsible for regulating PRL synthesis and secretion. However, the effect of dopamine (DA), which acts as an inhibitory factor on lactotroph function, also increases from birth to puberty, particularly in females. Nonetheless, the secretion of PRL remains higher in females compared to males. On the other hand, the observed sex differences in serum PRL levels during early postnatal development cannot be attributed to the influence of estradiol (E2). While serum E2 levels gradually increase after birth, only after 45 days of life do the disparities in E2 levels between females and males become evident. These observations collectively suggest that neither the maturation of hypothalamic DA regulation nor the rise in E2 levels can account for the progressive and sustained elevation in serum PRL levels and the observed sexual dimorphism during postnatal development. This review highlights the importance of recent discoveries in animal models that shed light on inhibitory mechanisms in the control of PRL secretion within the pituitary gland itself, that is intrapituitary mechanisms, with a specific emphasis on the role of transforming growth factor β1 and activins in PRL secretion.

Initially identified as an osmoregulatory factor in avian species, prolactin (PRL) was subsequently described in other vertebrates and received its name due to its stimulatory action on lactation in mammals [1]. Over the years, multiple biological functions attributed to this peptide hormone have been documented, usually extending beyond its originally designated role. PRL does not have a specific endocrine target. As the PRL receptor (PRL-R) is expressed in a wide array of tissues in mammals, PRL has many different targets and pleiotropic functions [2].

Nowadays PRL is known to be involved in several processes in mammals such as growth and development, reproduction, immunity, and behavior [3-6].

PRL synthesis and secretion take place in multiple tissues, including the central nervous system, immune system cells, and uterus [7], but the anterior pituitary gland is the primary source with lactotrophs being the local cells that synthesize and secrete this hormone. The major transcription factor required for PRL gene expression is *Pit1.* It plays an essential role in embryonic cell differentiation of thyrotrophs, somatotrophs, and lactotrophs. *Pit-1* regulates the expression of thyrotropin (TSH), growth hormone (GH), and PRL, and loss or mutation of this protein leads to hormone deficiency [8].

This review focuses on newly described mechanisms involved in the regulation of PRL secretion from lactotrophs during adulthood and postnatal development in murine animal models.

## Control of Prolactin Secretion

Lactotrophs are known for their constitutive activity in synthesizing and secreting PRL. Consequently, the primary regulatory mechanism from the hypothalamus is inhibitory, exerted mainly by dopamine (DA) [4, 9, 10]. Conversely, the principal stimulator of lactotroph functions is estradiol (E2). However, other PRL-releasing factors (PRFs) and PRL-inhibiting factors have been described, acting either directly on lactotrophs or indirectly via dopamine networks.

### Dopamine

DA is a catecholaminergic neurotransmitter that inhibits PRL synthesis and secretion *in vitro* and *in vivo*. Among the 5 distinct DA receptors described [11], the DA type 2 receptor (D2R) holds a central role in mediating this inhibitory action on lactotrophs. After agonist interaction D2R, coupled to pertussis toxin-sensitive Gi/Go proteins [12], triggers a rapid cellular response including an increase in potassium conductance and inactivation of voltage-gated calcium channels, leading to membrane hyperpolarization and subsequent inhibition of PRL release. Additionally, DA further suppresses adenylate cyclase activity, resulting in reduced cyclic adenosine monophosphate levels and decreased activation of protein kinase A. Consequently, this cascade of events culminates in the inhibition of PRL gene expression, contributing to the overall inhibitory control of PRL secretion [9].

Based on anatomical, histological, and functional studies performed specially in rats, 3 of all the dopaminergic pathways of the central nervous system have fundamental roles in the regulation of lactotroph functions: the tuberoinfundibular dopaminergic pathway (TIDA), the tuberohypophyseal dopaminergic pathway (THDA), and the paraventricular hypophyseal dopaminergic pathway (PHDA) (reviewed in [5, 9]).

The TIDA emerges as the most important neuron pathway in the inhibition of PRL. In this system, dopaminergic neurons located in the arcuate nucleus project their axons toward the median eminence releasing DA into the capillary network of the hypothalamic-pituitary portal system, reaching the anterior lobe of the pituitary gland. In the THDA, dopaminergic neurons from the arcuate nucleus extend their axons to the neurointermediate lobe, releasing DA that reaches lactotrophs via the intrahypophyseal portal system; the vessels connecting the posterior lobe with the anterior lobe. Lastly, the PHDA involves dopaminergic neurons projecting from the paraventricular nucleus of the hypothalamus to the pars intermedia of the pituitary gland. Although the PHDA’s primary function is to regulate the synthesis and secretion of melanocyte-stimulating hormone, DA released within this pathway can diffuse to the pars distalis and exert its inhibitory effect on lactotrophs [13-15].

### Other Hypothalamic Factors Involved in Prolactin Regulation

While DA represents the main inhibitory factor controlling the synthesis and release of pituitary PRL, additional PRL-inhibiting factors responsible for hypothalamic inhibition have been identified. Furthermore, the dopaminergic circuits are, in turn, regulated by a complex network of neurotransmitters, influencing DA release into the median eminence, thereby exerting an indirect effect on PRL secretion. These networks have previously been described in detail [5, 6, 9]. Briefly, several factors have been associated with the activation of TIDA neurons leading to the inhibition of PRL, such as acetylcholine, thyrotropin releasing-hormone (TRH), oxytocin, vasopressin, angiotensin II (AngII), vasoactive intestinal peptide (VIP), neuropeptide Y, calcitonin, bombesin, glutamate, and atrial natriuretic peptide, among others. Conversely, inhibitory effects on the TIDA neurons, leading to an indirect PRL stimulation, have been observed for serotonin, noradrenaline, opioid peptides, galanin, somatostatin, γ-aminobutyric acid (GABA), cholecystokinin, nitric oxide, and histamine. In addition, many of these factors (eg, GABA or somatostatin) are also secreted by neuronal terminals at the median eminence reaching their receptors on lactotrophs, leading to a direct inhibition of PRL secretion (reviewed in [5, 6]).

Among the PRFs, TRH plays an important role. The TRH neurons projecting to the median eminence have their cell bodies in the periventricular nucleus. TRH has been demonstrated to stimulate PRL release from lactotrophs in a dose-dependent manner, both *in vitro* and *in vivo* [16]. Suckling stimuli and estrogens increase TRH receptors in lactotrophs, thereby enhancing the PRL response to the tripeptide [17].

VIP, oxytocin, secretin, and angiotensin II were also proposed as hypothalamic PRFs when acting directly on lactotrophs (reviewed in [5]).

### Peripheral Organs

Undoubtedly, E2 is one of the most important stimulators of PRL secretion. In the pituitary gland, E2 increases the transcription of the PRL gene, decreases the expression of DA receptors, and induces lactotroph hypertrophy and the conversion of somatotrophs to lactotrophs [6]. E2 also acts on the hypothalamus, stimulating the circuits involved in the PRL release (eg, serotonin, oxytocin, VIP) and inhibiting the pathways involving PRL inhibitory factors [5].

Although E2 is the major stimulator of lactotroph functions, apoptotic [18] and antiproliferative [19] actions of the steroid have also been demonstrated in the anterior pituitary gland.

Studies performed in rats and somatolactotroph cell lines (GH3 and GH4) have demonstrated that E2 interferes the inhibition of lactotroph proliferation induced by insulin and insulin-like growth factor 1, sensitizing lactotrophs to the proapoptotic action of DA, and an E2 treatment increases the Bax/Bcl-2 ratio, enhancing the apoptotic rate of lactotrophs in the pituitary gland of ovariectomized rats (reviewed in [20, 21]). Although this evidence of the antiproliferative effects of E2 would seem to contradict its classic and well-described proliferative effects, it has been suggested that E2 induces a short latency antiproliferative action in the presence of growth factors, but a long-term mitogenic action mediated by the synthesis of local factors [22].

These opposite effects could also be related to the receptor subtype involved in the response. Accordingly, estrogen receptor α (ERα) activation induces lactotroph proliferation [23], G protein–coupled ER activation leads to PRL secretion *in vitro* and *ex vivo* [24], while E2 acting on lactotrophs through ERβ triggers antiproliferative effects. However, it is necessary to take into account the ERα/β ratio, as studies performed in pituitary cells from female rats demonstrated that the ERα/β ratio in normal (control) pituitaries is about 5:1, but it strongly increases after chronic E2 treatment, which induced development of a hyperplastic/adenomatous pituitary [19].

Although the role of E2 is well established, the effect of progesterone on PRL secretion is noticeably more complex. Several studies have documented varying effects on PRL secretion in response to progesterone: Some of them report enhancement whereas others suggest inhibition or no significant effect. These discrepancies could be partly attributed to the different sites of progesterone action (hypothalamus vs pituitary) [25], the type of receptor involved [26, 27], and even the hormonal environment, particularly E2 levels [28], among other contributing factors.

In addition to the gonads, regulatory factors from other peripheral organs can also affect PRL levels. For example, a dexamethasone treatment 2 hours before suckling stimulus completely blocked suckling-induced plasma PRL release in primiparous lactating rats, and the blockade of glucocorticoid receptors enhances PRL secretion [29]. However, a long-term increase in serum glucocorticoid levels decreases opioid-induced PRL secretion. These effects of glucocorticoids take place predominantly within the central nervous system affecting the TIDA neuron response [30]. Aside from the regulation of PRL secretion, glucocorticoids also stimulate the differentiation of somatotrophs suppressing that of lactotrophs in the fetal rat pituitary gland [31].

Furthermore, it is important to emphasize that, despite PRL lacking the classic negative feedback described for all other anterior pituitary hormones, PRL is still regulated by negative feedback. PRL itself provides an afferent signal through the median eminence in a process known as short-loop feedback, stimulating hypothalamic dopamine synthesis and turnover [32, 33], promoting DA secretion into the pituitary portal blood (reviewed in [6]).

### Intrapituitary Regulation of Lactotroph Function

In the anterior pituitary of mammals, paracrine communication among different pituitary cell populations and autocrine loops have been previously described. More than 100 compounds have been identified that have one or more paracrine or autocrine loops [34, 35]. In this regard, lactotrophs synthesize and secrete several peptides and growth factors that exert considerable influence over their own functionality (autocrine regulation). Interestingly, most of them are mediators of E2 action on lactotrophs, such as galanin, VIP, TGFα, and epidermal growth factor, leading to increased PRL secretion, and endothelins acting as an inhibitory autocrine loop of estrogen action [35, 36].

Gonadotroph-lactotroph is perhaps the paracrine regulation most studied in the pituitary. First evidence arises from observations that a gonadotropin-releasing hormone (GnRH) pulse unexpectedly triggers PRL release in lactotrophs lacking GnRH receptors [37]. Since then, several peptides have been identified in gonadotrophs as potential candidates for exerting paracrine influence on lactotrophs [37-39]. Among them, AngII has received particular attention [40, 41]. AngII colocalizes with follicle-stimulating hormone and luteinizing hormone in the secretory granules of gonadotrophs. Following GnRH stimulation, AngII is secreted, and on binding to its specific type 1 receptors (AT1) expressed in lactotrophs, it triggers the release of PRL [41, 42]. The first PRL-releasing effect of Ang II *in vivo* is observed at age 20 days in both sexes, without sexual differences, and it increases with age [43].

## Control of Prolactin Secretion During Postnatal Development

The serum profiles of several pituitary-secreted hormones present variations throughout postnatal development that are primarily attributed to the maturation of mechanisms involved in the control of the hypothalamic-pituitary axis.

During the first 3 weeks of life, the proportion of pituitary cells containing PRL and the amount of PRL storage in the gland are low. Then, these levels progressively increase with age until adulthood both in female and male rats [44, 45]. Interestingly, the profile of serum PRL levels during postnatal development exhibits a sexual dimorphism from birth, where female rats display higher PRL levels compared to age-matched males.

The postnatal maturation of the dopaminergic network cannot explain the gradual increase in serum PRL levels observed during the first weeks of life, nor the sex differences observed in this profile.

Dopaminergic neurons are initially identified in the periventricular nucleus on embryonic day (E) E12 in rats, in the arcuate nucleus on E14, and then are detected throughout the hypothalamus on E18. By E20, terminals from TH-positive neurons are evident in the median eminence, while the dopaminergic THDA and PHDA systems become functional by postnatal day 4 (P4) [46, 47]. This is consistent with the finding that catecholamines were first detected in the median eminence around the third to fifth postnatal day [48]. Subsequently, the dopaminergic inhibition of PRL [49] and the lactotroph responsiveness to this inhibition gradually increase until reaching puberty [50]. Blockage of D2R leads to a significant rise in serum PRL levels as early as P3, and this response progressively intensifies until age 35 days [51].

Sexual dimorphism in the development of dopaminergic systems involved in controlling PRL secretion is evident even at early stages. On E16, male rats exhibit a higher number of hypothalamic dopaminergic neurons compared to females. However, in females, these neurons present a larger size and increased DA content at this time [47]. Furthermore, Ojeda et al [52] described a sexual dimorphism in the *in vivo* response of PRL to the blockade of DA receptor after P15, being higher in females, and this effect becomes more pronounced after the third week of life. Then, it was demonstrated that the dopaminergic inhibition of PRL release increases from birth to puberty and that this inhibition is more pronounced in females even before birth.

Still, serum PRL levels gradually and continuously increase during postnatal development, with a sexual dimorphism by which females present higher levels of the hormone. Therefore, the next question is whether this phenomenon is attributable to the maturation of the mechanisms responsible for PRL release during the early weeks of life.

Interestingly, it was demonstrated that E2 is unable to increase PRL in rats younger than 24 days [51]. It was first suggested that this was related to the immaturity of the ERs through the hypothalamic-pituitary axis at this time [53]. However, it is important to note that during the first weeks of life, serum E2 remains tightly bound to the alpha fetoprotein, which prevents its interaction with the receptors and the subsequent initiation of the signaling pathway for PRL release. This serum protein is found at high concentrations in maternal blood, in the amniotic fluid, in fetal blood, and also in newborn serum [54]. Its concentration gradually decreases during the first 4 weeks of life in both sexes [55]. Furthermore, despite the rise in estrogen secretion from the maturing follicles observed in female ovaries, the presence of alpha fetoprotein during the first weeks of life determines that the sex difference in serum E2 levels appears only after 40 days of life [56]. Therefore, the PRL-releasing effect of E2 also cannot explain the sexual dimorphism observed in the profile of serum PRL levels during the first month of life.

Regarding hypothalamic PRL-releasing factors, the first assays were carried out by Karanth et al [57] using an *in vitro* model of the hypothalamic-pituitary axis. The authors observed that the hypothalamic PRL-releasing activity increases steadily from P7 to P56 in male rats. In addition, another group described that the PRL-releasing activity from the posterior pituitary is low before P20 and steadily increases from P30 to P90 [58].

Among the factors involved in these processes, an *in vivo* TRH stimulation can release TSH and PRL as early as the day of birth in both sexes, and this effect increases slightly as the animal matures, without sex differences [59]. However, the hypothalamic TRH content increases from birth to P14, but then gradually decreases to reach adult values [60]; even so, PRL continues increasing after P14.

Mechanisms involved in the serotonin-induced release of PRL are more complex. In adulthood, they are involved in the estrogen-induced release of PRL, in the PRL-releasing effect of opioids, in the afternoon surge of PRL, in the suckling-induced rise of PRL in lactating rats, and stress. However, it was demonstrated that serotonin agonists are unable to stimulate PRL secretion until P12 [59], reflecting that these stimulatory mechanisms mature after the second week of life.

All these antecedents indicate that neither the progressive increase in PRL secretion during postnatal development nor the sex differences observed in hormone secretion are related to a gradual decrease in the hypothalamic inhibitory activity or a gradual rise in stimulatory mechanisms.

In 1987, Chen [45] conducted a series of assays that revealed the inherent capacity of the pituitary gland to augment the production of PRL, even when isolated from hypothalamic regulation and peripheral organs. By using the reverse hemolytic plaque assay with isolated dispersed cells from rat anterior pituitaries, Chen demonstrated that at P5, only 5% of all anterior pituitary cells are secreting lactotrophs. This proportion gradually increases with age but presents at a sex-specific rate, reaching 37% of total pituitary cells in the adult male, and 54% in the age-matched females in proestrus. Therefore, these results indicate that the basal PRL release increases during postnatal development, even in the absence of hypothalamic or peripheral regulation. These results also suggest that local intrapituitary factors could be involved in the regulation of PRL secretion during postnatal development and contribute to the observed sexual differences.

## New Players on the Board

In addition to the main regulators (DA and E2) and the several intrapituitary factors described that hold a key role in the local regulation of lactotroph function, recent attention has been directed toward the involvement of two members of the transforming growth factor β (TGFβ) family: TGFβ1 and activins.

### Transforming Growth Factor β1 System

The effect of TGFβ1 on PRL secretion and lactotroph functions has been deeply studied in adult mice and rats as well as in cell lines [61-63]. In this regard, the cytokine was proposed as a potent inhibitor of PRL release and lactotroph proliferation (reviewed in [64]). Additionally, TGFβ1 was suggested as a mediator of the DA-inhibitory effect on lactotrophs as, on the one hand, DA increases the gene expression of TGFβ1 and TGFβ1 type II receptor in lactotrophs [65], and on the other hand, DA stimulates TGFβ1 biological activity [66, 67], resulting in the inhibition of lactotroph cell proliferation both *in vivo* and *in vitro*. Interestingly, this effect of DA is countered by the addition of a TGFβ1-neutralizing antibody [65].

Moreover, the decreased pituitary TGFβ1 activity observed in prolactinomas (in animal models and humans), coupled with the reduced expression of different components of the TGFβ1 system, has suggested a potential role of TGFβ1 in prolactinoma development [66, 68-71]. Indeed, it has been demonstrated that a pharmacological treatment with mimetic peptides of thrombospondin 1, an activator of TGFβ1, was able to restore pituitary TGFβ1 levels and counteract prolactinoma development. This finding introduces a novel therapeutic strategy to handle prolactinomas that are unresponsive to current DA-agonist treatments [71-73].

Interestingly, the inhibition of TGFβ1 on PRL release displays sex differences. The expression of most of the components involved in the TGFβ1 system and the pituitary levels of the active cytokine are increased in male pituitaries compared to females. This stronger inhibition of lactotroph function described in male pituitaries could be related to the sexual dimorphism observed in the development of prolactinomas in animal models [71, 74].

Recently, our group described the involvement of TGFβ1 in the regulation of PRL secretion during early postnatal days in rats, as well as its contribution to the observed sex differences [75]. Through an extensive characterization of the pituitary TGFβ1 system during postnatal development, we described that on P11, females exhibit the highest levels of active TGFβ1, concomitant with elevated protein expression of phospo-Smad3 specifically localized in lactotrophs, and increased messenger RNA (mRNA) expression of TGFβ1 target genes. These parameters decrease with age in this sex. Thereby, in females, the steady increase in PRL secretion during early postnatal life displays an inverse correlation with pituitary active-TGFβ1 levels, as well as with mRNA expression of several components of the TGFβ1 system, such as TGFβ receptors, TGFβ-latent protein 1 (Ltbp1), and local TGFβ1 activators such as thrombospondin 1 (Tsp1). On the contrary, male pituitaries present lower levels of active TGFβ1 at P11; these levels are maintained relatively constant up to P23, and increase toward adulthood, without any correlation with serum PRL levels.

These findings highlight that the regulatory role of TGFβ1 on PRL secretion is specific to females, suggesting that this mechanism is involved in the sexual dimorphism observed in the profile of serum PRL levels during postnatal development [75].

### Activins

Activins belong to the TGFβ superfamily, existing as homodimers or heterodimers composed of the β subunits βA and βB. These proteins act as paracrine and/or autocrine factors in the pituitary, and their local action is antagonized by inhibins (heterodimers of β and α subunits), follistatins, and betaglycan [76]. Although inhibins are locally synthesized in the pituitary, the predominant source of inhibins that reach the pituitary comes from the gonads [77].

Initially, activins were identified as factors that stimulate the release of follicle-stimulating hormone from gonadotrophs, but it was later discovered that activins inhibit PRL secretion and lactotroph cell proliferation [78, 79].

As members of the TGFβ1 family, the canonical signaling pathway of activins involves SMADs proteins. However, it has been described that activin inhibition of PRL gene expression also involves *Pit-1* inhibition through the p38MAPK pathway [80].

Recent research has uncovered sexual dimorphism in the activin inhibition of PRL secretion in adult mice. Male pituitaries present higher mRNA expression of activin subunits, suggesting a greater bioavailability of activins compared to females, and thus, a stronger activin inhibitory effect on the lactotroph population in this sex [81].

The relevance of the activins’ inhibitory action on lactotrophs became evident when it was suggested that a decrease in their biological activity could play a role in the development of prolactinomas [81]. In this context, and compared with normal pituitaries, prolactinomas from 2 different animal models present a decreased expression of activins and a reduced proportion of lactotrophs expressing activin type 1 receptor. It was concomitant with a significant decrease in the proportion of lactotrophs expressing p-p38MAPK, and consequently, with an increase in *Pit1* expression. This sequence of events could contribute to prolactinoma development [81]. Furthermore, when female mice from these animal models of prolactinomas undergo ovariectomy before age 2 months, the development of tumors does not occur. The removal of the primary source of inhibins, the ovaries, results in a sharp increase in the expression and the biological function of pituitary activins, recovering the inhibitory action and helping to counteract prolactinoma development [82].

Apart from the inhibitory role exerted by activins on lactotrophs in adult mice, recent findings have pointed out the involvement of activin-inhibitory action in regulating PRL secretion during early postnatal development as well [83]. Data from developing rats indicate that the pituitary activin expression and biological function develop in sex-specific patterns during early postnatal age. In females, the pituitary expression of the activin subunits, *Inhba* and *Inhbb*, is maximum at P11 and then decreases with age. The same outline is observed with the gene expression of activin type 1 and 2 receptors, and the protein expression of activin receptor type 1 specifically in the lactotroph population. Moreover, activin biological activity, measured as the p-p38MAPK expression in lactotrophs, is also maximum at P11 in females. The proportion of lactotrophs expressing p-p38MAPK decreases with age, facilitating a gradual rise in the gene expression of Pit-1 [83]. Then, activin expression and biological activity negatively correlate with serum PRL levels in females during early postnatal development.

Conversely, in males, the pituitary expression of *Inhba* and *Inhbb* at P11 is significantly lower than in age-matched females. For *Inhba* the expression increases with age, reaching values even higher than those observed in females at P45. Then, in males, the pituitary expression of activins and their receptors do not show any correlation with PRL levels [83].

These results suggest that activins play an important role in the regulation of lactotroph functions during early postnatal days. Furthermore, the regulatory influence of activins on PRL secretion is sex specific, exclusive to females. This implies that pituitary activin function contributes to the sexual dimorphism observed in serum PRL levels during postnatal development.

## Concluding Remarks

**Figure 1.**
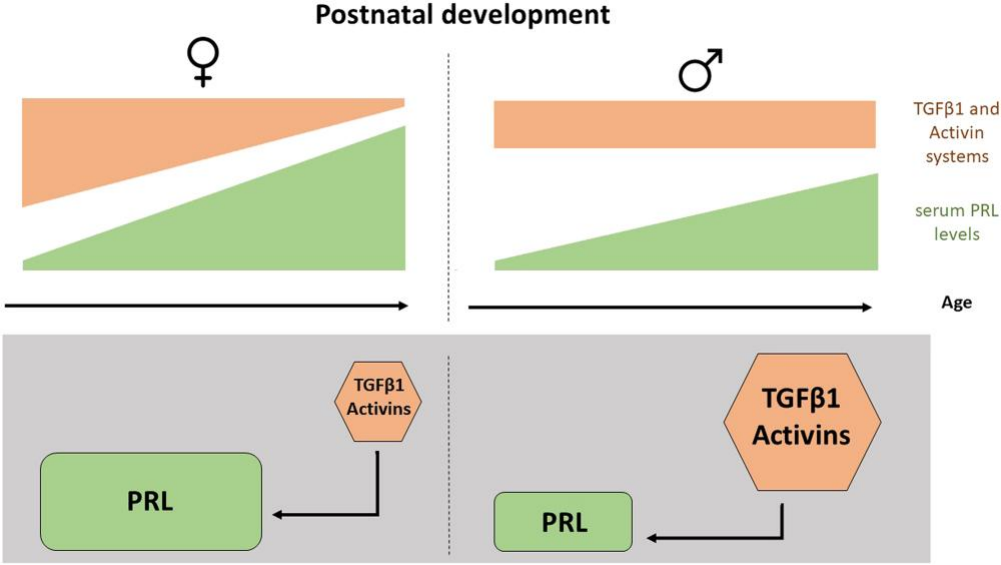
The regulation of PRL by TGFβ1 and activins is sex- and age-specific. Upper panel: Serum prolactin levels exhibit a gradual rise in both male and female rats from birth to adulthood, with females consistently displaying higher levels compared to age-matched males. Female pituitaries present stronger expression and biological action of TGFβ1 and activins at early postnatal days, decreasing with age and displaying an inverse correlation with serum PRL levels only in this sex. Bottom panel: These systems, and their inhibitory action on PRL secretion, become stronger in adult male pituitaries.

Numerous studies have outlined the importance of DA and E2, among other hypothalamic factors and peripheral hormones, in the control of lactotroph function and PRL release. However, the maturation of these systems cannot fully explain the PRL pattern, nor its sex differences observed during early life. Over the past decade, substantial progress has been made in murine animal models uncovering new players on the board that potentially explain the sexual dimorphism observed. Several studies have shown the importance of the local inhibitory action exercised by TGFβ1 and activins in the physiology and pathology of the anterior pituitary gland (eg, prolactinomas). More recently, these mechanisms were suggested to play a role in the sex differences observed in PRL secretion in adulthood. In addition, data obtained from developing rats support the concept that TGFβ1 and activins are also involved in the control of PRL secretion during postnatal development. Moreover, being sex specific, the participation of these local intrapituitary inhibitors affects the sexual dimorphism observed in the profile of PRL secretion during the first months of life (Fig. 1).

While our understanding of the intricate control of PRL secretion remains incomplete, the characterization of intrapituitary factors that inhibit lactotrophic functions makes them attractive as potential therapeutic targets for conditions characterized by deregulation of PRL secretion. It is indispensable to translate these findings from animal models to human pathologies, as they may hold promising therapeutic prospects, particularly in the context of treating prolactinomas.

## Data Availability

Data sharing is not applicable to this article as no data sets were generated or analyzed during the current study.

## Abbreviations

AngII: angiotensin II
D2R: dopamine type 2 receptor
DA: dopamine
E: embryonic day
E2: estradiol
ERα: estrogen receptor α
GH: growth hormone
GnRH: gonadotropin-releasing hormone
mRNA: messenger RNA
P: postnatal day
PHDA: paraventricular-hypophyseal dopaminergic pathway
PRF: prolactin-releasing factor
PRL: prolactin
PRL-R: prolactin receptor
TGFβ1: transforming growth factor β1
THDA: tuberohypophyseal dopaminergic pathway
TIDA: tuberoinfundibular dopaminergic pathway
TRH: thyrotropin-releasing-hormone
TSH: thyrotropin
VIP: vasoactive intestinal peptide
